# Potent Fc Receptor Signaling by IgA Leads to Superior Killing of Cancer Cells by Neutrophils Compared to IgG

**DOI:** 10.3389/fimmu.2019.00704

**Published:** 2019-04-11

**Authors:** Arianne M. Brandsma, Sina Bondza, Mitchell Evers, Rosanne Koutstaal, Maaike Nederend, J. H. Marco Jansen, Thies Rösner, Thomas Valerius, Jeanette H. W. Leusen, Toine ten Broeke

**Affiliations:** ^1^Laboratory of Translational Immunology, University Medical Center Utrecht, Utrecht, Netherlands; ^2^Ridgeview Instruments AB, Vänge, Sweden; ^3^Department of Immunology, Genetics and Pathology, Uppsala University, Uppsala, Sweden; ^4^Division of Stem Cell Transplantation and Immunotherapy, Department of Internal Medicine II, Christian-Albrechts-University, Kiel, Germany

**Keywords:** IgA, immunotherapy, neutrophil, ADCC, cancer, Fc alpha receptor I, CD89, signaling

## Abstract

Antibody therapy of cancer is increasingly used in the clinic and has improved patient's life expectancy. Except for immune checkpoint inhibition, the mode of action of many antibodies is to recognize overexpressed or specific tumor antigens and initiate either direct F(ab′)_2_-mediated tumor cell killing, or Fc-mediated effects such as complement-dependent cytotoxicity (CDC) and antibody-dependent cell-mediated cytotoxicity/phagocytosis (ADCC/P) after binding to activating Fc receptors. All antibodies used in the clinic are of the IgG isotype. The IgA isotype can, however, also elicit powerful anti-tumor responses through engagement of the activating Fc receptor for monomeric IgA (FcαRI). In addition to monocytes, macrophages and eosinophils as FcαRI expressing immune cells, neutrophils are especially vigorous in eliminating IgA opsonized tumor cells. However, with IgG as single agent it appears almost impossible to activate neutrophils efficiently, as we have visualized by live cell imaging of tumor cell killing. In this study, we investigated Fc receptor expression, binding and signaling to clarify why triggering of neutrophils by IgA is more efficient than by IgG. FcαRI expression on neutrophils is ~2 times and ~20 times lower than that of Fcγ receptors FcγRIIa and FcγRIIIb, but still, binding of neutrophils to IgA- or IgG-coated surfaces was similar. In addition, our data suggest that IgA-mediated binding of neutrophils is more stable compared to IgG. IgA engagement of neutrophils elicited stronger Fc receptor signaling than IgG as indicated by measuring the p-ERK signaling molecule. We propose that the higher stoichiometry of IgA to the FcαR/FcRγ-chain complex, activating four ITAMs (Immunoreceptor Tyrosine-based Activating Motifs) compared to a single ITAM for FcγRIIa, combined with a possible decoy role of the highly expressed FcγRIIIb, explains why IgA is much better than IgG at triggering tumor cell killing by neutrophils. We anticipate that harnessing the vast population of neutrophils by the use of IgA monoclonal antibodies can be a valuable addition to the growing arsenal of antibody-based therapeutics for cancer treatment.

## Introduction

Antibody therapy for cancer treatment is increasingly used in the clinic after the successful introduction of rituximab over two decades ago, which is an IgG monoclonal antibody (mAb) directed against CD20 expressed on B cells. This success has been followed by the development of many IgG-based mAbs for cancer treatments and has considerably improved treatment outcome. These mAbs can employ direct working mechanisms through their F(ab′)_2_ domains by interfering with target function or inducing complement-dependent cytotoxicity (CDC) after binding of C1q to clustered Fc domains on the tumor cell surface. Next to CDC, binding of the IgG Fc domain to Fcγ receptors (FcγR) expressed on immune cells can elicit antibody dependent cell-mediated cytotoxicity/phagocytosis (ADCC/P). For antibody therapy in humans, it remains difficult to assess the contribution of each working mechanism for different mAbs, but *in vivo* experiments have exposed an important contribution of Fc receptor-mediated ADCC/P (1, 2). In addition, the role of FcγR in humans has been further demonstrated by genetic polymorphisms of FcγR that influence clinical outcome of mAb therapy (3).

All the current therapeutic mAbs for cancer are based on the IgG isotype. Reasons for this include its natural prevalence in the body, long half-life of IgG, and the substantial amount of fundamental and biotechnological knowledge of this isotype. IgG mAbs that trigger ADCC/P are described to activate NK cells by FcγRIIIa and monocytes/macrophages by the various activating FcγRs they express. Activating FcR signal via ITAMs (Immunoreceptor Tyrosine-based Activating Motifs), either in their cytoplasmic domain or via the FcR-associated gamma chain. Upon antibody binding and crosslinking of FcR, ITAMs will first bind and activate Lyn and/or Fyn tyrosine kinases, depending on the immune cell. Subsequently, phosphorylated ITAMs will recruit and activate Syk followed by the activation of SOS, Ras, Rac, PKC, PI3K, and finally ERK or MAP kinase, inducing gene transcription of cytokines, inflammatory mediators, microbicidal enzymes, activation of the cytoskeleton, all together leading to ADCC, phagocytosis, cell migration, and degranulation. These pathways are comparable between different activating Fc receptors for different Ig isotypes (4). Recent discoveries advocate that other isotypes, like IgA and IgE, are also promising options for tumor treatment (5, 6).

IgA directed against tumor cells has been proven to be effective *in vivo*, which largely depends on the presence of FcαRI, the myeloid Fc receptor for monomeric IgA (7–11). FcαRI is expressed by innate immune cells, including monocytes, macrophages, Kupffer cells, eosinophils, and neutrophils (12, 13). Neutrophils are the most abundant immune cells in the body, representing up to 70% of circulating leukocytes. They migrate through and surveil tissues, including malignant tumors, and can mediate antibody-induced anti-tumor effects (14, 15). Neutrophils are superior at eliminating IgA-opsonized tumor cells compared to IgG using EGFR, CD20, HER2 and HLA II as targets in *in vitro* studies (7, 9, 10, 16–20). In addition, *in vivo* studies using intraperitoneal tumor models indicate that neutrophils are recruited to the peritoneum upon IgA treatment (unpublished data). Neutrophils can exert several effector functions, including phagocytosis, degranulation, ROS production and NET formation (21). These effector mechanisms are found not to be causal for killing of opsonized tumor cells. Recently, a new neutrophil effector mechanism, termed trogoptosis, has been characterized (22, 23). Trogoptosis is target cell death initiated by antibody-FcR-triggered trogocytosis (24) executed by stimulated neutrophils and has also been described as ADCT (antibody-dependent cell-mediated trogocytosis). A similar process has been postulated to be executed by macrophages but this requires more time (25).

To improve antibody therapeutics, it would be promising to mobilize the vast number of neutrophils with IgA to help eradicate tumor cells from the body. In the current study we investigated the underlying mechanisms of the superior IgA-mediated tumor cell killing by neutrophils in comparison to IgG. First, IgA and IgG were compared in classical ^51^Cr release assays using three clinically used targets to verify published observations. To visualize the IgA-mediated killing by neutrophils, we performed live-cell imaging in a similar setup. An explanation for the strong ability of IgA to efficiently induce killing by neutrophils could be a correlating difference in the expression of FcγRs and FcαRI. Therefore, the quantitative FcR expression level on neutrophils and their binding capacity to IgG- and IgA-coated or -opsonized surfaces were explored. Antibody Fc domain binding to FcR facilitates its ITAM phosphorylation and ultimately the phosphorylation and activation of ERK. Therefore, we also investigated the dynamics of p-ERK in neutrophils after interaction with IgA- and IgG-bound surfaces, and found significant differences.

## Materials and Methods

### Reagents, Antibodies

**Figure 1:**
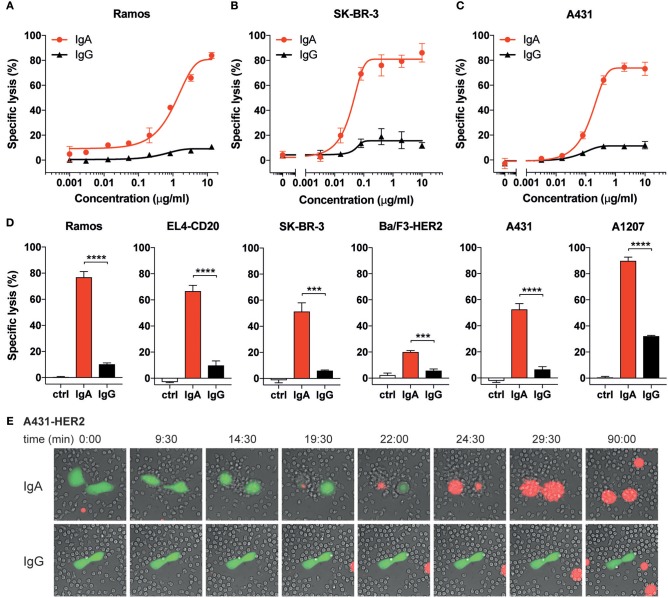
IgA mediates higher tumor cell lysis than IgG with human neutrophils. **(A–D)** Specific lysis of tumor cells by isolated human neutrophils (E:T = 40:1) using a 4 h ^51^Cr release assay. Anti-CD20 Abs were used for Ramos and EL4-CD20, anti-HER2 Abs were used for SK-BR-3 and Ba/F3-HER2, anti-EGFR Abs were used for A431 and A1207 target cells. **(A–C)** Specific lysis of indicated target cells using a broad titration range of IgA and IgG antibodies. **(D)** Antibody concentrations were identical (10 μg/mL) for IgG and IgA antibodies against all target cells except for Ramos (13.3 μg/mL) and A1207 cells (1 μg/mL). Ctrl indicates condition without antibody (or 0.001 μg/mL IgG1-anti-CD20 for Ramos cells). One representative graph is shown for *n* = 4–6 independent experiments with different healthy donors. *p* < 0.001: ^***^, *p* < 0.0001: ^****^, unpaired Student's *t* test. **(E)** Stills from live cell microscopy of adhered A431-HER2 cells (calcein labeled) and neutrophils in the presence of 5 μg/mL anti-HER2 IgA2 or IgG (Trastuzumab) and TO-PRO-3 (red fluorescence), time in minutes, image acquisition every 30 s for 1.5 h.

Antibodies: to target human CD20, rituximab (Roche), anti-CD20-IgA1 (invivogen, hcd20-mab6), or in-house made mAbs (26) were used. For HER2, anti-HER2 IgG1 antibody trastuzumab (Roche), anti-HER2 IgA1 or IgA2 antibody was used and for EGFR, anti-EGFR IgG1 antibody cetuximab (Merck) or anti-EGFR IgA2 was used. Anti-HER2 or EGFR IgA's were made as described before (11). 3G8 F(ab′)_2_ (anti-FcγRIII) was generated by pepsin protease procedure of purified 3G8 from hybridoma supernatant. Rabbit anti-ERK (9101S) and rabbit anti-p-ERK (9102S) both from Cell Signaling Technology, anti-β-Actin (Sigma-Aldrich, A2228), anti-rabbit IgG HRP (Santa Cruz, sc-2004), goat anti-mouse IgG HRP (Santa Cruz, sc-2005), Strep-Tactin HRP (Bio-Rad Laboratories, 16-10380) were used for Western blot detection. The Qifikit (DAKO) was used to determine the number of FcγRs on primary neutrophil isolates using anti-CD64 (clone 10.1, Serotec), anti-CD89 (clone A59, BD Pharmingen), anti-CD32a (clone IV.3, Stemcell Technologies), anti-CD16 (clone 3G8, Stemcell Technologies), and anti-CD32a/b (clone AT10, Santa Cruz Biotechnology or clone KB61, DAKO). Pharmacological compounds Wortmannin (Sigma-Aldrich), LY294002, and U0126 (Calbiochem) were used to inhibit ITAM signaling in ^51^Cr release assays. Calcein-AM (Life Technologies) was used to fluorescently label target cells or neutrophils according to the manufacturers protocol. TO-PRO^**TM**^-3 (molecular probes) was used at 1:1000 dilution to detect DNA that becomes accessible during live-cell imaging (Figure 1E, Videos S1, S2).

### Primary Neutrophils and Cell Lines

Neutrophil cell fraction was isolated from blood of healthy donors (in agreement with ethical committee of the Utrecht university medical center and after written informed consent from the subjects in accordance with the Declaration of Helsinki) using standard Ficoll/Histopaque density block gradient centrifugation (Ficoll-paque was from GE healthcare, ref. 17-1440-03, Histopaque-1119 was from Sigma-Aldrich ref. 11191) followed by lysis of the red blood cells in ammonium buffer (155 mM NH_4_Cl, 10 mM KHCO_3_, 0.037 mg/mL Na_2_EDTA, pH 7.4) for 10 min on ice. Neutrophils were preserved in complete medium (RPMI 1640 from Gibco supplemented with glutamine, 100 U/mL penicillin and 100 μg/mL streptomycin from Life Technologies) before use on the same day unless stated otherwise. EL4-CD20, Ba/F3-HER2, and A431-HER2 cells (ATCC) were generated by retroviral transduction as described before (18). Ramos, Daudi, SK-BR-3, A431 (ATCC), and the above-mentioned cells were cultured in complete medium at 37°C and 5% CO_2_.

### Live-Cell Imaging

For live-cell imaging, a Deltavision RT widefield microscope (GE Healthcare) equipped with a conditioned imaging chamber set to 37°C and 5% CO_2_ was used. Time-lapse imaging was performed using an Olympus 40×/1.35 NA (numerical aperture) oil immersion objective (Figure 1E, Videos S1, S2) or an Olympus 20×/0.75 NA objective (Figure S1, Videos S3–S5) and images were recorded on a Cascade II EM-CCD camera (Photometrics). For Videos S1, S2, the A431 cells were seeded in a 6-channel μ-slide (Ibidi) the day before calcein labeling and live cell imaging. Target cells in Videos S3–S5 were harvested and cytosolically labeled with calcein-AM and allowed to interact with primary neutrophils together with mAbs for up to 2 h in the 6-channel μ-slide. Image acquisition (30–60 s between frames) started as soon as possible upon addition of the mAbs. Imaging data was processed using SoftWoRx (AppliedPrecision) or Imaris (Bitplane).

### Human Neutrophil ADCC

ADCC with ^51^Cr-labeled target cells was described previously (18). Briefly, 1 × 10^6^ target cells were labeled with 100 μCi (3.7 MBq) ^51^Cr for 2 h in complete medium. After extensive washing, cells were adjusted to 10^5^/mL. Neutrophils, mAbs at various concentrations, medium, and 5,000 tumor cells per well were added to round-bottom microtiter plates (Corning Incorporated) using a maximum E:T = 40:1 ratio. When indicated, neutrophils were preincubated for 15 min at RT with inhibitors of signaling molecules before they were added to the plate. After 4 h of incubation at 37°C, ^51^Cr release was measured in counts per minute (cpm). The percentage of specific lysis was calculated using the following formula: % lysis = [(counts of sample–minimum release)/(maximum release–minimum release)] × 100. Target cells with neutrophils in complete medium or supplemented with 5% Triton X-100 (Roche Diagnostics) were used to determine minimum and maximum release, respectively.

### Binding of Neutrophils on Antibody-Coated Plastic Surface

For binding assays in 96-wells flat bottom maxisorp plates, wells were coated with 100 μL 10 μg/mL antibody in carbonate buffer (Sigma, C3041-50CAP) O/N at 4°C. Plates were washed with 100 μL complete culture medium, blocked for 1 h with 1% BSA and washed again with culture medium before adding the cells. Neutrophils were isolated from blood and labeled with 10 μM Calcein-AM in PBS at 37°C for 20 min. Cells were washed two times with complete medium and rested for 30 min at 37°C at a density of 3 × 10^6^ cells/mL. 100 μL neutrophil suspension was added per well and centrifuged gently (~50 × g) for 3 min. The plate was incubated at 37°C for 40 min. Fluorescence was measured in a Clariostar fluorescence scanner. After measurement, the plate was washed with 100 μL RPMI. Measurements were repeated every 2 washes and this was repeated at least 12 times.

### Bead Binding to Neutrophils (Rosettes)

The rosette assay using Dynabeads was adapted from a previously described protocol (27). Epoxy-Dynabeads (4.5 mm) were coupled to either anti-CD20 mAbs UMAB002 IgG1, UMAB002 IgA2, anti-HER2 mAbs trastuzumab (Herceptin) IgG1, anti-HER2 IgA2 (own production), or human serum albumin (Albuman^®^, Sanquin) following the manufacturer's instructions (Thermo Fisher Scientific). Labeling of the beads was checked with RPE-labeled anti-IgA (Southern Biotech, 2052-09), and PE-labeled anti-IgG (Southern Biotech, 2042-09). To allow bead binding, 50 μl 2 × 10^6^ cells/mL neutrophils in ice-cold PBS containing 0.5% BSA were pipetted in a round bottom 96-wells plate (Greiner) on ice. 50 μL 1 × 10^7^ beads/mL bead suspension in PBS containing 0.5% BSA was added to the cells. Beads were either coated with anti-CD20 or anti-HER2 IgG1 or IgA2. Plates were incubated on a shaker (800 RPM) at 4°C for at least 30 min. The plate was then incubated at 37°C for 10 min and immediately put on ice. After centrifugation and supernatant removal, the samples were fixed in 3% PFA in PBS containing 0,5% BSA for 15 min at RT. The fixed cell/bead mixture was transferred to a flat-bottom 96-wells plate, centrifuged (~50 × g) for 3 min and images were taken with brightfield microscopy (EVOSRXLCore) and analyzed using Adobe Photoshop using raster blocks of 11 × 11 cm. Cells were counted manually and the percentage of cells bound to 5 or more beads was calculated per image, for 3 images per condition.

### Real-Time Tracing of Antibody-Mediated Cell-Cell Interactions

Daudi cells were adhered in quarters A&C of LigandTracer Multidishes 2 × 2 (non-treated, Ridgeview Instruments AB) with the help of a biomolecular anchor molecule (SUNBRIGHT^®^ OE-040CS, NOF Corporation), essentially as previously described (28). Per immobilization spot, 400 μl BAM solution (4 mg/ml, dissolved in MQ water) was incubated for 1 h at room temperature and after removal of the BAM solution 400 μl cell suspension (1.5 × 10^6^ cells/ml in PBS) was added and cells were left to adhere for 40 min. Prepared cell dishes were kept in complete medium in the incubator and used for experiments the following day. For measuring antibody mediated cell-cell interactions, one of the immobilized Daudi cell spots was preincubated with 50 nM of either anti-CD20 IgG1 or IgA2 (own production, clone UMAB001) for 1 h in LigandTracer Green (Ridgeview Instruments AB) at room temperature. Antibody preincubation was done only for one of the compartments, the other half of the dish served as control for quantifying non-antibody mediated neutrophil interactions. To each half of the dish, 3 × 10^6^ calcein-labeled neutrophils from the same donor were added and binding to Daudi cells was recorded once every 75 s. After 1 h, neutrophils were removed and the remaining cell-cell complexes were followed for another hour to observe their stability. Binding slopes from the no antibody control were used to normalize the data for differences in signal height between experiments. The binding association between IgG1 and IgA2 was compared by the ratio of the binding slope, which was calculated with TraceDrawer 1.8 (Ridgeview Instruments AB). The stability of the formed cell-cell complexes is represented by the half-life, which was calculated from the dissociation rate constant k_d_ that was obtained by fitting a single exponential decay to the dissociation phase (TraceDrawer 1.8).

### Neutrophil Stimulation With Antibody-Coated Beads and Western Blotting

Neutrophils were brought to 1 × 10^8^ cells/ml in Hanks buffer. Per condition, neutrophils were stimulated with antibody-coated beads in a 1:4 = neutrophil:bead ratio at 37°C for the indicated time periods. After incubation, cells were lysed in Laemmli reducing sample buffer and boiled. SDS-PAGE (12%) was performed and gels were blotted on nitrocellulose membranes. For ERK/p-ERK detection, the membranes were first blocked in 4% ELK milk in TBST (TRIS buffered saline/0.1% Tween 20), washed in TBST and incubated with anti-ERK or anti-p-ERK both at 1:2500 in TBST containing 0.3% BSA at 4°C overnight. Then, blots were washed in TBST, blocked in 4% ELK milk in TBST for 1 h at RT and sequentially incubated with anti-rabbit IgG HRP (1:2500) and Strep-Tactin HRP (1:5000) in 1% ELK milk in TBST for 1 h at RT. Blots were sequentially washed with TBST and PBS followed by ECL-based detection in a BioRad Gel Doc. For the β-actin immuno staining, the HRP from the former detection was first destroyed by 24 h incubation at 4°C in PBS containing 0.1% Tween 20, 0.6% sodium azide, and 0.6% H_2_O_2_. This solution was refreshed at least three times during incubation. After this, the blots were washed with TBST, blocked for 1 h at RT in 4% ELK milk in TBST followed by 2 h incubation with anti-β-Actin (1:2000) in 1% milk in TBST. The blots were then washed with TBST and incubated for 1 h with goat anti-mouse IgG HRP (1:5000) in 1% milk and further processed for detection like described above.

### Statistical Analysis

Graphs represent mean ± SD. Statistical analysis was performed by using unpaired Students *t*-tests or ANOVA with Tukey's multiple comparison test, *p* < 0.05 were considered as statistically significant.

## Results

### IgA Mediates Superior Neutrophil Cytotoxicity on Tumor Cells Compared to IgG

We and others have demonstrated that IgA-opsonized tumor cells, in contrast to IgG, are efficiently killed by freshly isolated neutrophils (7, 9, 10, 16–20). To verify previous observations, we performed ^51^Cr release assays using three targets (CD20, HER2, and EGFR) and freshly isolated unstimulated neutrophils from healthy donors (Figure 1). The mAbs were first titrated in a broad concentration range and this shows that, although the antibody concentration for IgA and IgG for detectable lysis is similar, the highest maximum amount of lysis is achieved by IgA (Figures 1A–C). We further inventoried this with two cell lines per target, confirming that the IgA isotype consistently outperforms IgG in tumor cell lysis by primary unstimulated neutrophils (Figure 1D). The magnitude of lysis did vary between mAbs, cell lines and neutrophil donors, but the superiority of IgA was found to be highly constant in this setting.

To visualize the process of antibody-mediated tumor cell killing by neutrophils, we performed live-cell imaging. In the presence of anti-HER2 IgG (Trastuzumab), A431-HER2 cells did not elicit a clear recruitment of unstimulated primary neutrophils and only minimal interaction between A431-HER2 and neutrophils was observed (Figure 1E lower panel and Video S2). In contrast, neutrophils rapidly respond to A431-HER2 in the presence of anti-HER2 IgA (Figure 1E upper panel and Video S1). The fast reaction of neutrophils suggests a swarming effect toward the calcein-labeled tumor cell and involves numerous interactions which seem reminiscent of trogocytosis (23). Only in the presence of IgA, the tumor cells succumb to these events within 30 min as indicated by the fluorescence of a DNA-binding dye. The killing process of IgA-opsonized cells was also visualized for EGFR- and CD20-expressing target cells and demonstrated similar dynamics between neutrophils and tumor cells. Several lysis events are observed with IgA (Figures S1A,B, Videos S3–S5) whereas with IgG no lysis could be recorded (data not shown). These data confirm the superiority of IgA over IgG in neutrophil-mediated killing of tumor cells for three different targets and seems to involve the recently described trogoptosis as an antibody-dependent cytotoxic working mechanism of neutrophils.

### Differential FcR Expression on Neutrophils Does Not Explain IgA Superiority

**Figure 2:**
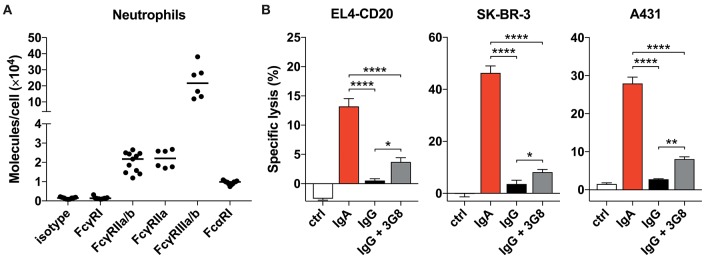
Blocking FcγRIIIb only marginally improves tumor cell lysis. **(A)** Quantitative expression of FcγR and FcαRI on human neutrophils as analyzed by flow cytometry (Qifikit), *n* = 6–11 healthy donors. **(B)** 3 h ^51^Cr release assays using EL4-CD20 with anti-CD20 IgA2 or IgG1 (rituximab), SK-BR-3 with anti-HER2 IgA1 or IgG1 and A431 with anti-EGFR IgA2 or IgG1, all at 10 ug/mL. 3G8 F(ab′)_2_ fragments (1 ug/mL final concentration) were added 15 min prior to start of the ADCC assays. Statistics: one-way ANOVA with Tukey's multiple comparison test, *p* < 0.05: ^*^, *p* < 0.01: ^**^, *p* < 0.0001: ^****^. Ctrl indicates no antibody added.

To find a possible explanation for the robust IgA-dependent tumor cell killing, we quantified FcαRI expression on unstimulated primary neutrophils. A relatively high expression of FcαRI would possibly explain the stronger activation of neutrophil effector mechanisms after engagement with IgA-opsonized cells. However, our analysis of the number of FcR molecules per neutrophil demonstrated that the expression of FcαRI is actually ~2-fold lower than that of FcγRIIa, the activating FcγR on neutrophils (Figure 2A). FcγRI and FcγRIIIa are not detectable on unstimulated neutrophils (29) and the GPI-linked FcγRIIIb is expressed at least 10-fold higher than FcγRIIa and FcαRI. The high constitutive expression of FcγRIIIb is unique for neutrophils as this is not observed on monocytes or NK cells (Figure S2) and other granulocytes (29).

Neutrophils do not express an ITIM (Immunoreceptor Tyrosine-based Inhibitory Motif)- containing FcγR that could counteract signaling by activating FcγRs. Therefore, we continued by investigating the potential role of FcγRIIIb that has been reported to interfere with Fc-mediated effects, although it is a GPI-linked protein and cannot signal on its own (30–32). Blocking FcγRIIIb with 3G8 mAb F(ab′)_2_ in a ^51^Cr-release assay did, however, only marginally improve IgG-mediated lysis of tumor cells (Figure 2B) and did not change with 10 times higher 3G8 concentration (data not shown). For all three antibodies tested, IgA outperformed IgG even after FcγRIIIb blockade. From these data we conclude that neither the FcR expression levels nor a major role for FcγRIIIb in inhibiting FcγRIIa function are causal for the discrepancy between IgA- and IgG-mediated killing by primary neutrophils.

### Neutrophils Have Similar Binding Characteristics to IgA- or IgG-Bound Surfaces

**Figure 3:**
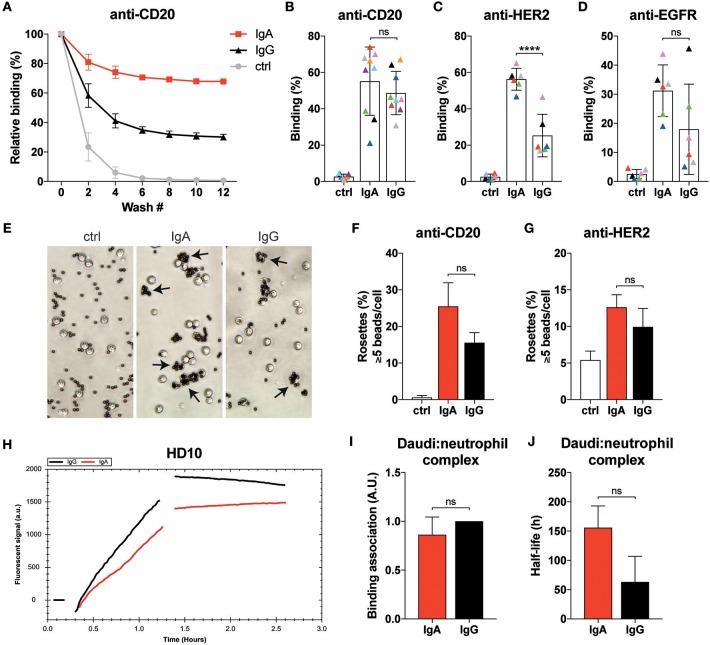
Neutrophils display similar binding characteristics to IgA- or IgG-associated surfaces. **(A)** Example of relative binding of a donor of which calcein labeled neutrophils were allowed to associate to anti-CD20 IgA2 or IgG1 (clone UMAB001) coated 96 well plate. Remaining calcein fluorescence was measured after repeating wash steps. Calcein fluorescence before the first wash was set 100%. Ctrl indicates that no antibody was used during coating. **(B–D)** As in **A**, but data of 6–9 different donors at wash 10 are combined per target being anti-CD20 IgA2 or IgG1 (clone UMAB001) or anti-HER2 IgA2 or IgG1. Each color indicates data from the same donor. Statistics: two-way ANOVA with Tukey's multiple comparison test, *p* < 0.0001: ^****^. **(E)** Examples images of neutrophils binding to albumin, anti-CD20 IgA, or IgG coated Dynabeads (ratio beads:neutrophils = 5:1). Rosettes are defined as cells binding 5 or more beads. **(F,G)** Quantification of rosettes of anti-CD20- or anti-HER2-coated Dynabeads with neutrophils. One representative of *n* = 3 individual experiments is shown. Differences were not significant according to one-way ANOVA with Tukey's multiple comparison test. **(H)** Binding traces for calcein labeled neutrophils binding to anti-CD20 IgA- (red) or IgG- (black) opsonized Daudi cells. One representative of *n* = 5 experiments is shown. **(I)** Binding association and **(J)** average half-life of the Daudi:neutrophils complex pooled from 5 different donors. Statistics: paired Student's *t*-test.

Efficient IgA-induced killing by neutrophils could also be driven by stronger binding to its FcR compared to IgG. The binding affinities of monomeric IgA to FcαRI and monomeric IgG to FcγRIIa and FcγRIIIb are in the low affinity range with reported *K*_a_ of < 10^7^ M^−1^ and therefore believed to be short-lived (29, 33). Stable binding is only achieved when sufficient avidity is established by an increase in valency from the multiple interactions of FcRs to antibody-bound surfaces. Therefore, we interrogated the binding characteristics of primary neutrophils to antibody-coated or -opsonized surfaces. Unstimulated primary neutrophils from healthy donors were labeled with calcein, allowed to bind antibody-coated surfaces and subjected to several wash steps while monitoring calcein fluorescence in the wells (Figures 3A–D). Often, higher binding of neutrophils to IgA-coated surfaces was measured (Figure 3A), but, depending on donor and mAb, the reverse was also found (Figures 3B,D). Only for the HER2 target a consistent better binding to the IgA isotype was observed with the used donors.

We continued by performing rosette assays where neutrophils are exposed to IgA- or IgG-coated beads (Figures 3E–G). The example images shown in Figure 3E reveal that more neutrophils bind IgA-coated beads. Further analysis with either anti-CD20- or anti-HER2-coated beads showed a trend for more rosettes and thus better binding with IgA compared to IgG, but this did not reach statistical significance (Figures 3F,G).

Binding dynamics of neutrophils to opsonized target cells was also explored with LigandTracer technology (28). Calcein-labeled primary neutrophils were first allowed to engage anti-CD20 IgA- or IgG-opsonized Daudi cells as illustrated by the increase in fluorescent signal (Figure 3H). After removal of the unbound neutrophils, the remaining neutrophils were monitored further in time to study the stability of the neutrophil-Daudi interaction. This approach confirmed our previous observations that there is no significant difference between IgA- and IgG-mediated binding of neutrophils (Figure 3I). We did, however, notice an increased stability of the Daudi:neutrophil complex (Figure 3J, Figure S3) when IgA was used, although significance is not reached. Taken together, binding of neutrophils to IgA bound surfaces is at least as good as with IgG despite the lower expression of FcαRI compared to the FcγRs.

### IgA Elicits Stronger ITAM Signaling in Neutrophils Than IgG

**Figure 4:**
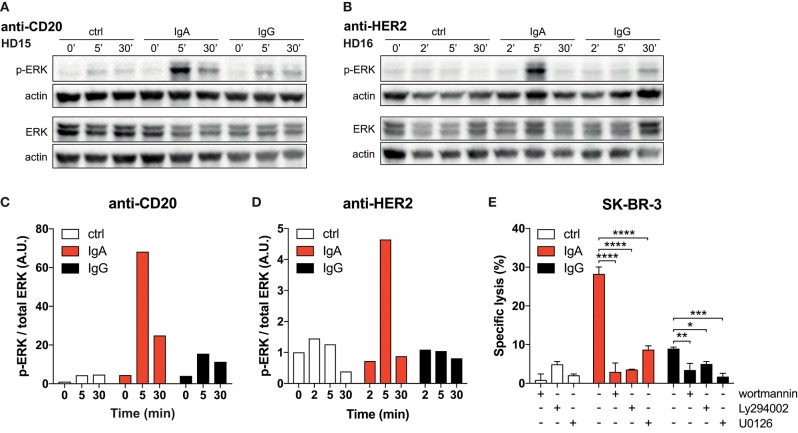
Strong p-ERK signal in neutrophils elicited by IgA. **(A,B)** Time-dependent increase and decrease of p-ERK signal in neutrophils after exposure to anti-CD20 or anti-HER2 IgA2- or IgG1-coated Dynabeads. Actin detection was performed on the same blot after destroying the p-ERK or total ERK signal. **(C,D)** Quantification of signaling defined as the ratio of normalized p-ERK/actin over normalized total ERK/actin. Data was normalized against the 0 min time point. **(E)** 4 h ^51^Cr release assays using SK-BR-3 with anti-HER2 IgA2 or IgG1, both at 1 ug/mL. Where indicated, pharmacological inhibitors of signaling factors were included (wortmannin at 0.5 μM, Ly294002 and U0126 both at 20 μM). Statistics: two-way ANOVA with Tukey's multiple comparisons test *p* < 0.05: ^*^, *p* < 0.01: ^**^, *p* < 0.001: ^***^, *p* < 0.0001: ^****^.

Thus far, a clear mechanism that accounts for the superior IgA- vs. poor IgG-mediated killing by neutrophils has not been identified. For neutrophils to exert their effector functions after FcR binding, ITAM signaling is required. Therefore, we measured the magnitude of signaling in neutrophils after binding to antibody-coated surfaces. Analysis by immunoblotting revealed a rapid and strong phosphorylated ERK (p-ERK) signal after 5 min when neutrophils were allowed to interact with IgA-coated beads, while IgG-coated beads induced only a weak p-ERK signal (Figures 4A–D). These conditions were repeated with several neutrophil donors for both anti-CD20 and anti-HER2 antibodies, demonstrating a reproducible effect on the p-ERK signal (Figures 4A–D, Figures S4A–C).

**Figure 5:**
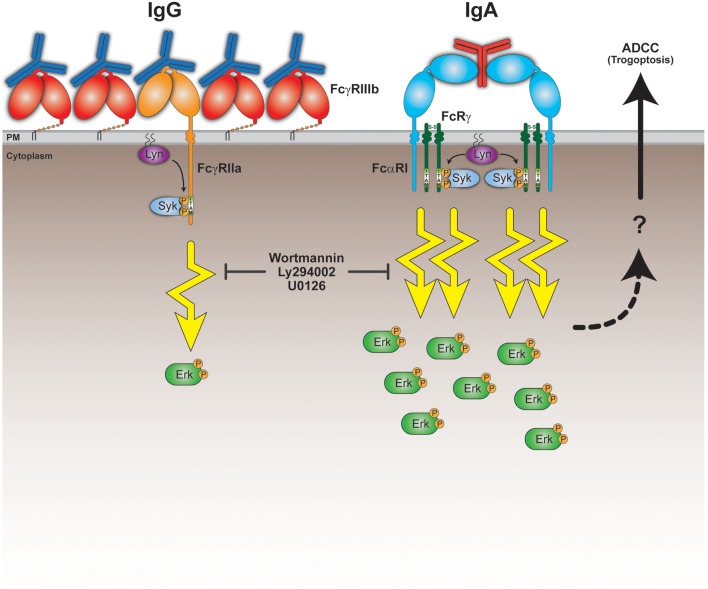
Model for superior IgA-mediated tumor cell killing by neutrophils. Neutrophils express the low affinity Fc receptors FcγRIIIb, FcγRIIa, and FcαRI. Both FcγRIIa and FcαRI are activating Fc receptors, because upon ligand (antibody Fc domain) binding, clustering and recruitment of kinases (e.g., Lyn), they signal through their ITAM domains and elicit cellular effector functions. The relatively high FcγRIIIb expression on neutrophils could possibly interfere with the activating capacity of FcγRIIa by competing for Fc domain binding and preventing proper formation of signaling platforms and/or organization within the lipid bilayer. This results in a poor ability of FcγRIIa to initiate sufficient signaling in neutrophils. Despite the low FcαRI expression on neutrophils, they can still engage clustered IgA Fc domains to a similar degree as IgG Fc domains bind the FcγRs. IgA can, however, bind FcαRI bivalently resulting in more stable binding and recruitment of in total 4 ITAMs. This scenario would initiate a robust ITAM signaling necessary for activating effector functions, including trogoptosis to eliminate tumor cells. This is further illustrated by the signaling inhibitors wortmannin, Ly294002 and U0126 that prevent tumor cell lysis *in vitro*. The question mark refers to yet unclear processes involving signaling, intracellular Ca^2+^, actin-myosin contraction, and immune cell-tumor cell interactions that lead to ADDC by neutrophils (trogoptosis) (23).

To confirm the crucial role for the robust FcαRI signaling, we tested the PI3K inhibitors wortmannin and Ly294002 and the MEK1/2 inhibitor U0126, which both act upstream of ERK in the signaling cascade. The presence of these pharmacological inhibitors in ^51^Cr-release assays resulted in a significant decrease in tumor cell lysis (Figure 4E). This quantitative difference in signaling and its requirement for efficient antibody mediated killing leads to a model where FcαRI signaling is stronger than FcγRIIa signaling in neutrophils, which can explain the efficient killing of IgA-opsonized tumor cells (Figure 5).

## Discussion

FcR-bearing immune effector cells have a prominent role in antibody therapies for cancer treatment by engaging Fc domains of IgG-opsonized tumor cells. Due to the IgG based format of these therapeutics, only FcγR-mediated ADCC, ADCP, and/or ADCT mechanisms can be employed by immune cells. Our data confirms that unstimulated neutrophils are very poor at eliciting lysis of tumor cells through the IgG-FcγR axis. Yet, it could be very propitious if the vast number of neutrophils in the body could be recruited for tumor eradication. In agreement with previous work, we demonstrated for three targets (CD20, EGFR, and HER2) that the IgA isotype is very potent at triggering tumor cell killing by unstimulated neutrophils. Former studies have compared this to IgG-mediated killing by NK cells and demonstrated that the maximal IgA-mediated killing by neutrophils is often higher (7, 10, 18). NK cell-mediated killing by IgG does seem to be more efficient at lower antibody concentrations compared to IgA-induced lysis by neutrophils. Visualization of the IgA-mediated killing process by neutrophils did not reveal signs of phagocytosis, but suggests a killing mechanism that includes frequent and vigorous interactions between neutrophils and IgA-opsonized tumor cells, most likely involving trogocytosis. These observations agree with trogoptosis, a trogocytosis-based mechanism that has been postulated for IgG-mediated killing by stimulated neutrophils (23) and recently submitted work on IgA (Treffers et al. submitted). Neutrophils have been described to display a swarming behavior toward sites of inflammation or tissue damage in which LTB4 secretion is an important molecule (34–36). In our live-cell experiments (Video S2), migration of neutrophils toward the IgA-opsonized tumor cell resembles this swarming phenotype that precedes tumor cell death. If such a process could be established in malignant tumors by IgA mAbs, it might break the immune tolerant tumor microenvironment and drive a robust anti-tumor response.

In our quest for the explanation of the superior IgA-mediated tumor cell killing by neutrophils, we investigated its binding dynamics to antibody-bound surfaces, the role of FcγRIIIb and its FcR expression levels. Unexpectedly, FcαRI expression by neutrophils is ~2-fold lower than that of FcγRIIa. The FcαRI expression level measured on neutrophils does, therefore, not provide a simple explanation for the robust IgA-elicited killing by neutrophils in comparison to the poor IgG-mediated killing. Blocking of the highly expressed GPI-bound FcγRIIIb during a ^51^Cr-release assay also does not restore IgG-induced tumor cell lysis to the level achieved with IgA. Still, the abundant expression of FcγRIIIb could interfere by preventing optimal FcγRIIa organization at the plasma membrane required for efficient ITAM signaling and triggering of neutrophil effector function. Another reason for the efficient IgA-mediated killing could be a better qualitative and quantitative IgA-FcαRI interaction by neutrophils. We conducted several binding studies using IgA- or IgG-bound surfaces and even opsonized cells as platforms for unstimulated primary neutrophils to bind to (Figure 3). Although a trend of better binding for IgA was observed, no clear significant differences for all of the tested targets were found. The very consistent superiority of IgA-mediated lysis does not correlate with the similarity we see in binding to IgA or IgG of neutrophil from different donors. Thus, the characterized binding dynamics do not provide a satisfactory answer for the effectiveness of IgA-induced killing by primary neutrophils.

As binding characteristics cannot explain the poor IgG-mediated killing, we reasoned that it might not be the binding itself that is the crucial factor, but rather the events that happen after binding. Indeed, the magnitude of FcR signaling within neutrophils after exposure to IgA on p-ERK level is much stronger than for IgG (Figures 4A–D). It was confirmed in Figure 4E that inhibitors of the ITAM signaling are very effective in preventing IgA-mediated tumor cell lysis (Figure 4E). Therefore, very strong ITAM signaling upon FcαRI engagement explains the potent neutrophil effector functions. The relatively low FcαRI expression on neutrophils, but yet similar binding to IgA and its powerful IgA-induced signaling suggests a fundamental difference in the manner of FcR engagement. FcαRI is able to interact with IgA in a 1:1 or a 2:1 (FcαRI:IgA) stoichiometry (Figure 5) (37). A bivalent binding of FcαRI to IgA would result in a stronger association, which is supported by our observation that the half-life of IgA-mediated binding tends to be longer compared to IgG (Figure 3J). Moreover, FcγRIIa can only signal through one ITAM located in its cytoplasmic tail, whereas FcαRI in a 2:1 stoichiometry would deploy four ITAMs by the FcαRI-associated FcRγ-chains resulting in much stronger signaling (Figure 5). It would be interesting to test this model by comparing monovalent vs. bivalent IgA-FcαRI binding. This requires challenging engineering of IgA molecules in which the crucial residue(s) for FcαRI binding (38) are mutated in one of the two heavy chains.

Therapeutic IgA for cancer treatment has not yet entered clinical trials but there are promising *in vivo* results in mouse models (7–11). It could, therefore, be a valuable addition or alternative for patients that do not respond or have become resistant to IgG therapy. Depending on the location and tumor type, the rigorous activation of neutrophils by IgA could be a very welcome alternative for IgG, particularly since neutrophils are the most abundant type of leukocyte in the body. Next to this, we and others have demonstrated that simultaneous engagement of FcγRs and FcαRI enhances tumor cell killing (18, 39). Great progress has been made in cancer treatment since the modulation of immune checkpoint molecules was included in immunotherapy (40, 41). This has primarily been focused on eliciting T-cell immunity against the tumor. Likewise, myeloid cells express checkpoint molecules as well. The most prominent example is the myeloid-restricted signal regulatory protein alpha (SIRPα) that transduces the “don't eat me” signal when bound to the ubiquitously expressed CD47 (42). Recent work has demonstrated that blocking SIRPα-CD47 axis potentiates IgG antibody therapies (43), but even more so enhances the therapeutic potential of IgA monoclonals [(44) Treffers et al., submitted].

In conclusion, we have demonstrated that unstimulated primary neutrophils are able to kill IgA-opsonized tumor cells efficiently in contrast to IgG. Our current model (Figure 5) suggests that the strong induction of FcαRI signaling is crucial for this process. Taken together, these promising developments support a solid base for exploring the possibilities of IgA therapeutics further and improve future treatment of cancer.

## Data Availability

All datasets generated for this study are included in the manuscript and/or the Supplementary Files.

## Author Contributions

JL, TtB, AB, and TV: conceptualization; TtB, JJ, AB, SB, ME, RK, MN, and JL: methodology; AB, TtB, SB, ME, MN, JJ, and RK: formal analysis; TR: providing crucial reagents; TtB, AB, SB, JJ, and RK: investigation; TtB and JL: writing—original draft; TtB, AB, ME, JJ, MN, RK, SB, JL, and TV: writing—review and editing; AB and TtB: visualization; TtB and JL: supervision; TtB, TV, and JL: funding acquisition.

### Conflict of Interest Statement

SB is employed by Ridgeview Instruments AB. JL is a scientific founder and shareholder of TigaTx. The remaining authors declare that the research was conducted in the absence of any commercial or financial relationships that could be construed as a potential conflict of interest.

## Supplementary Material

Figure S1 Stills from live-cell microscopy of anti-CD20 IgA and anti-EGFR IgA facilitated tumor cell killing by PMNs. **(A)** Calcein labeled EL4-CD20 cells or **(B)** A431 cells were imaged together with unstimulated human PMNs in the presence of anti-CD20 IgA1 **(A)** or anti-EGFR IgA2 **(B)**. Killings of target cells are indicated by the colored circles with corresponding time points above the images. Effector:target ratio for anti-CD20 IgA1 **(A)** was 15:1 and **(B)** for anti-EGFR IgA2 10:1.

Figure S2 Quantitative expression of FcγR and FcαRI on primary human monocytes **(A)** or NK cells **(B)** of *n* = 6–11 healthy donors using flow cytometry (Qifikit).

Figure S3 Remaining individual donors from which PMN binding dynamics to anti-CD20 IgG1or IgA opsonized Daudi cells were measured using ligand tracer technology.

Figure S4 **(A,C)** All five individual donors for p-ERK induction, showing the p-ERK and ERK blots. **(B)** Quantification of the anti-CD20 blots, by dividing the p-ERK signal over the ERK signal. The 0 min time point was set to 1. A.U. = arbitrary units, 3 donors are combined. Statistics: two-way ANOVA with Tukey's multiple comparison test. Data are mean + SEM (note: all other graphs in paper are mean + SD), *p* < 0.05: *.

Video S1 Anti-HER2 IgA2 (5 μg/ml) mediated killing of calcein labeled adhered A431-HER2 cells by unstimulated primary human neutrophils. Tumor cell lysis is visualized by the red fluorescence of the DNA dye TO-PRO^TM^-3.

Video S2 Live-cell imaging of adhered A431-HER2 cells in the presence of Anti-HER2 IgG1 (5 μg/ml, trastuzumab), TO-PRO^TM^-3, and unstimulated primary human neutrophils.

Video S3 EL4-CD20 were labeled with calcein and live-cell imaged in the presence of anti-CD20-IgA1 (5 μg/ml) and unstimulated primary human neutrophils, E:T = 15:1.

Videos S4,5 Live-cell imaging of calcein labeled A431 cells in suspension together with anti-EGFR IgA2 (5 μg/ml) and unstimulated primary human neutrophils, E:T = 10:1.
