# Supplementary material for: Potent Fc Receptor Signaling by IgA Leads to Superior Killing of Cancer Cells by Neutrophils Compared to IgG
Source: Front Immunol. 2019 Apr 11;10:704. doi: 10.3389/fimmu.2019.00704 (PMC6470253; doi:10.3389/fimmu.2019.00704)
Supplement: Figure S1 — Stills from live-cell microscopy of anti-CD20 IgA and anti-EGFR IgA facilitated tumor cell killing by PMNs. (A) Calcein labeled EL4-CD20 cells or (B) A431 cells were imaged together with unstimulated human PMNs in the presence of anti-CD20 IgA1 (A) or anti-EGFR IgA2 (B). Killings of target cells are indicated by the colored circles with corresponding time points above the images. Effector:target ratio for anti-CD20 IgA1 (A) was 15:1 and (B) for anti-EGFR IgA2 10:1. [file Data_Sheet_1.pdf]

Figure S1

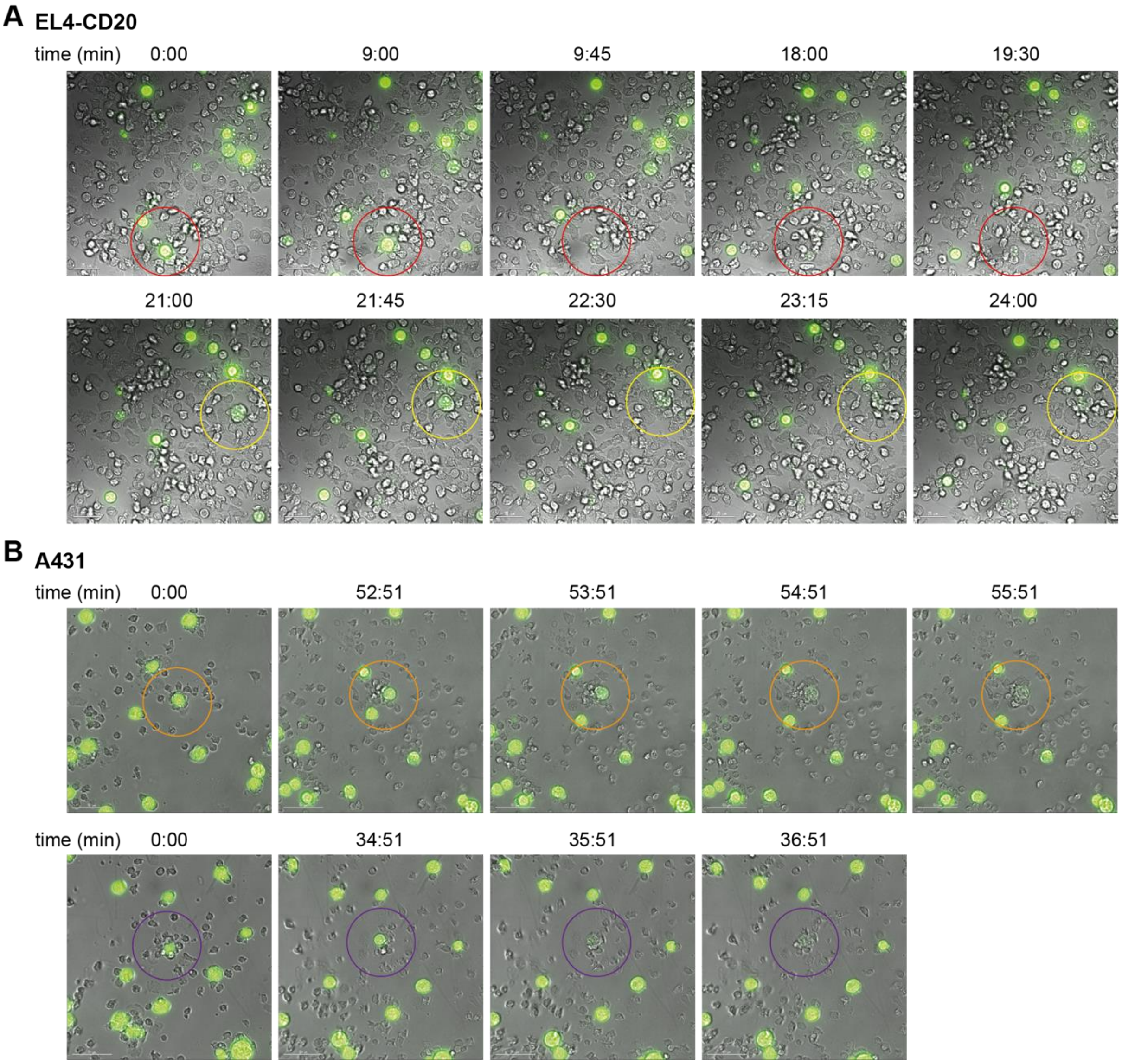

Figure S1. Stills from live-cell microscopy of anti-CD20 IgA and anti-EGFR IgA facilitated tumor cell killing by PMNs. (A) Calcein labeled EL4-CD20 cells or (B) A431 cells were imaged together with unstimulated human PMNs in the presence of anti-CD20 IgA1 (A) or anti-EGFR IgA2 (B). Killings of target cells are indicated by the colored circles with corresponding time points above the images. Effector:target ratio for anti-CD20 IgA1 (A) was 15:1 and (B) for anti-EGFR IgA2 10:1.

Figure S2

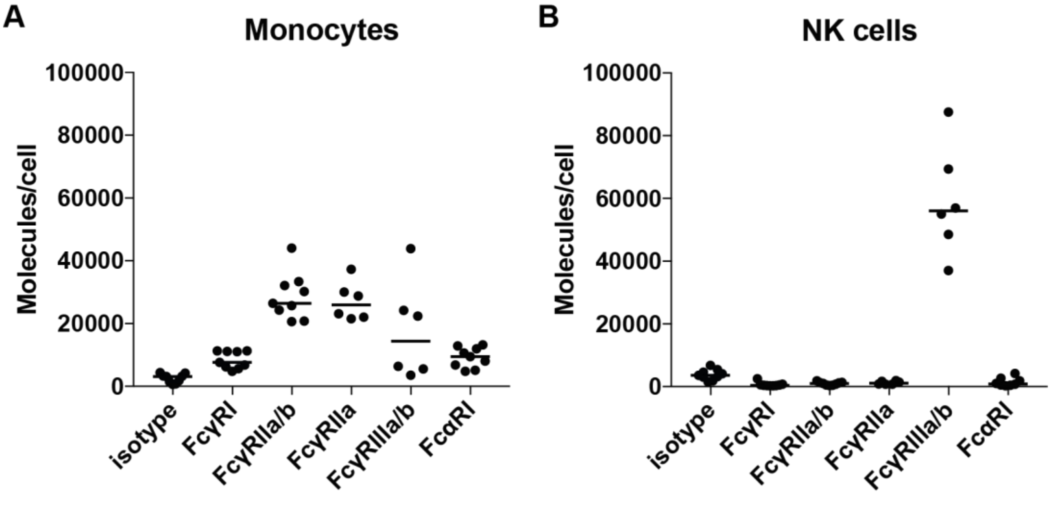

Figure S2. Quantitative expression of FcγR and FcαRI on primary human monocytes (A) or NK cells (B) of n=6-11 healthy donors using flow cytometry (Qifikit).

Figure S3

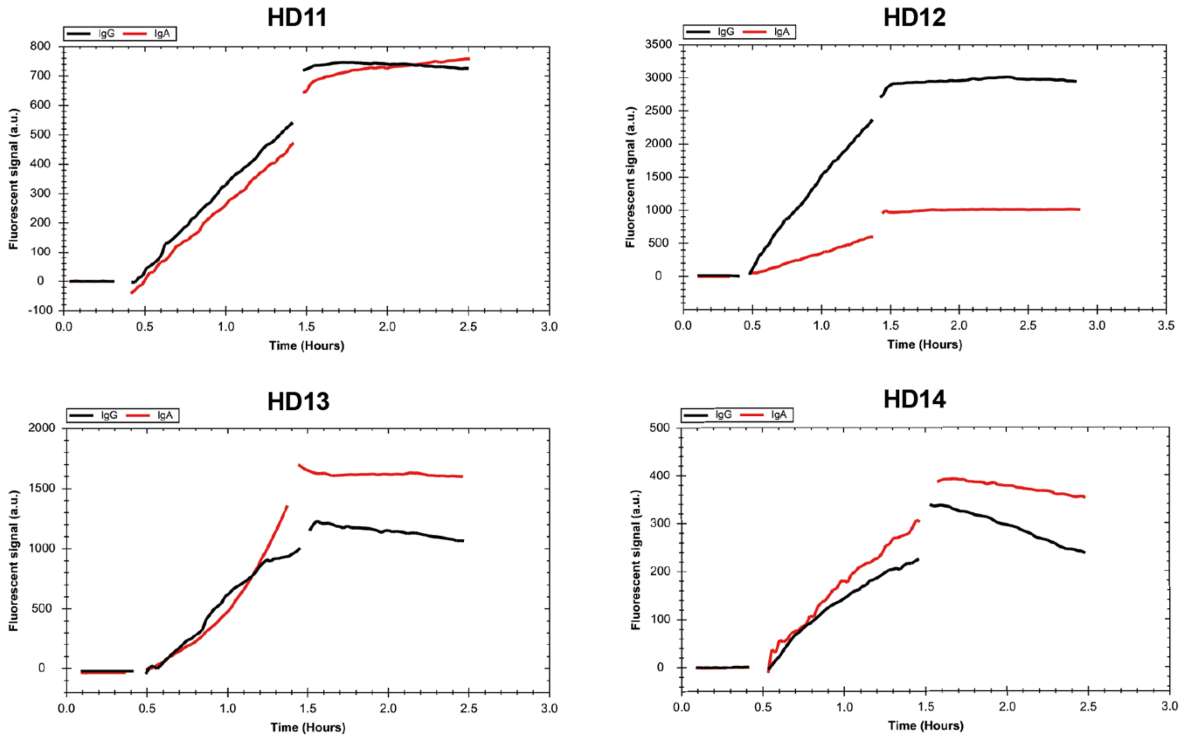

Figure S3. Remaining individual donors from which PMN binding dynamics to anti-CD20 IgG1or IgA opsonized Daudi cells were measured using ligand tracer technology.

Figure S4

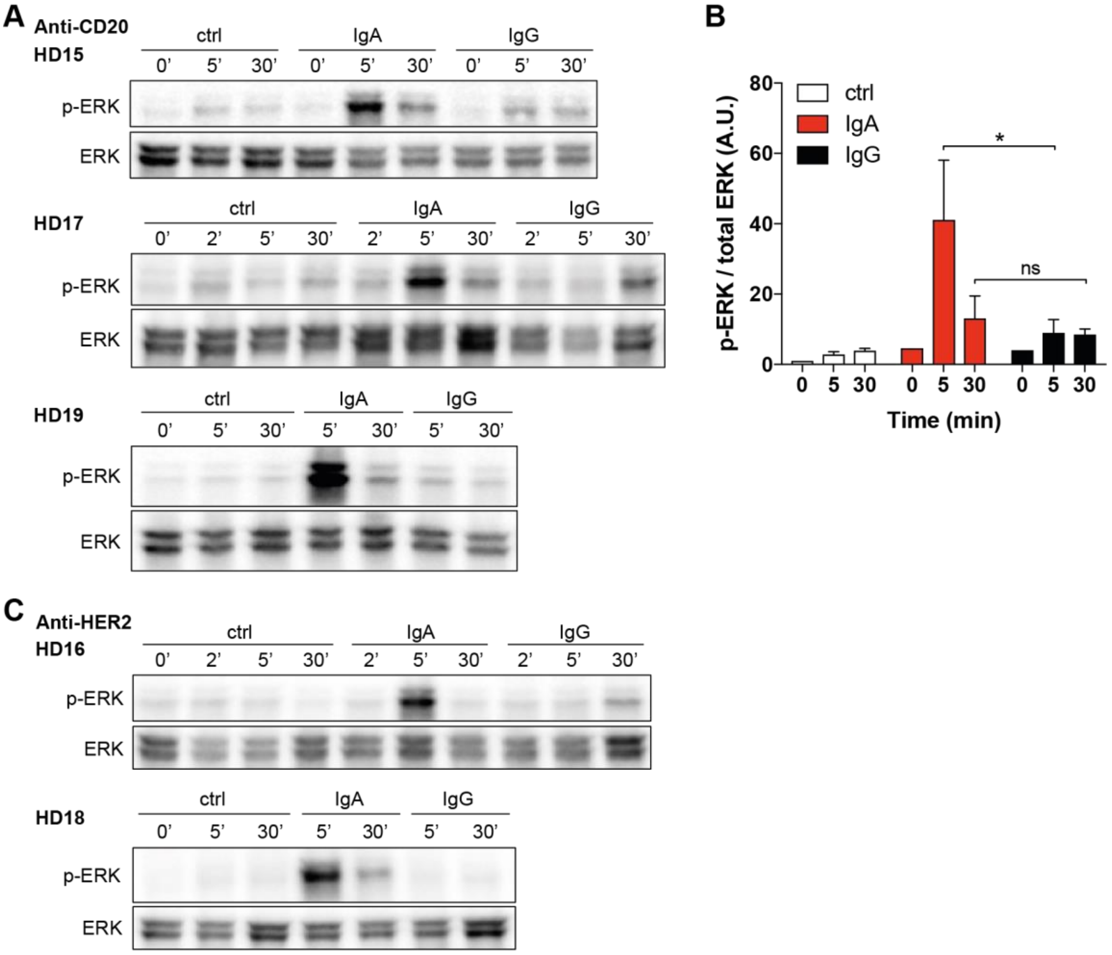

Figure S4. (A and C) All five individual donors for p-ERK induction, showing the p-ERK and ERK blots. (B) Quantification of the anti-CD20 blots, by dividing the p-ERK signal over the ERK signal. The 0 min time point was set to 1. A.U. = arbitrary units, 3 donors are combined. Statistics: two-way ANOVA with Tukey's multiple comparison test. Data are mean + SEM (note: all other graphs in paper are mean + SD), $p < 0.05$: *.
